# Threats and Interventions on Wellbeing in Asylum Seekers in the Netherlands: A Scoping Review

**DOI:** 10.3389/fpsyt.2022.829522

**Published:** 2022-04-01

**Authors:** Ferdy Pluck, Roelof Ettema, Eric Vermetten

**Affiliations:** ^1^Department of Psychiatry, Leiden University Medical Centre, Leiden, Netherlands; ^2^Research Group Personalized Integrated Care, University of Applied Science Utrecht, Utrecht, Netherlands; ^3^Amethist Addiction Care, Almere, Netherlands; ^4^Arq Psychotrauma Expert Group, Diemen, Netherlands; ^5^Department of Military Mental Health Care (MGGZ), Ministry of Defense, Utrecht, Netherlands

**Keywords:** asylum seeker, mental health, psychotrauma, interventions, stressors

## Abstract

**Background:**

Most asylum seekers experience stress, not only due to the reason for fleeing and their travel but also due to their compulsory stay in the asylum seeker center in the Netherlands and the asylum procedure. This often leads to self-medication and addiction which causes lower self-esteem and lower quality of life. Adverse life events, forced migration, and prolonged asylum procedures, in addition to the complexity of the acculturation process, can all contribute to higher levels of psychopathology.

**Objective:**

What are the threats to wellbeing in terms of mental health, psychosocial, and addiction problems, and what are the effective interventions for wellbeing for asylum seekers in asylum seeker centers in the Netherlands, reported in the literature?

**Method:**

Following the descriptive nature of the research question and the need for identifying knowledge gaps, an overview of existing knowledge was created by executing a scoping review on influencing factors on the mental health of asylum seekers. The Neuman system model was used as a guiding framework to understand the complexity of the issues this population experience and to identify the stressors and the factors which cause the imbalance and also the disease.

**Results:**

The literature review resulted in 26 articles that met the criteria for inclusion. The threats included the influence of staying in the environment of an asylum seekers center, drug abuse among asylum seekers, health-care professionals and employees who do not detect or underestimate the underlying suffering of asylum seekers, and frequent relocations of asylum seekers. The two assessment instruments used were the Rapid-Assessment-Response method (RAR method) and the Health Information Assessment Tool Asylum Seeker tool (HIATUS tool). Finally, the five interventions were identified: therapy for asylum seekers diagnosed with post-traumatic stress disorder (PSTD), art therapy, education focused on prevention as intervention, cultural interview, and mindspring.

**Conclusion:**

The knowledge on identifying and reducing threats, assessment, and treatment interventions for asylum seekers living in an asylum seekers center found in the literature provided perspectives on improving their wellbeing. The great diversity of cultural aspects and continuous changes in the number and origin of refugees in the Dutch asylum seekers centers disrupted the continuity of care.

## Highlights

- Treatment possibilities on mental health conditions in asylum seekers.
- The vulnerability of asylum seekers.
- Poor mental health leads to lower outcomes on well-being.

## Introduction

Currently, in Europe, the asylum seeker centers get overcrowded due to increased refugee flows (1). In recent years and nowadays in the Netherlands, the majority of asylum seekers come from Syria. On top of that, Dutch asylum seeker centers are now faced with a sudden rise in refugees from Afghanistan due to the recent change in the political situation in Afghanistan (2).

In September 2021 in the Netherlands, 30% of first-time asylum seekers came from Afghanistan, 29% Syria, 8% Turkey, 5% Somalia, 5% Yemen, 3% Algeria, and 2% unknown nationality (1). In the year prior, 30% of first-time asylum seekers came from Syria, 12% Turkey, 11% Afghanistan, and 6% Algeria (1).

Asylum seekers in Dutch asylum seekers centers are often heavily burdened with mental health problems (3). Mental health problems and self-medication were triggered by war, political instability, poor economy, and worsened during travel to the Netherlands (4). Self-medication often led to addiction, lower quality of life, and generalized displeasure (4). How asylum seekers perceived and coped with illness in terms of their culture of origin was often unknown by professionals providing mental health care in refugee camps in the Netherlands (3).

Numerous reasons were identified why asylum seekers initially leave their home country, including by war, religion, beliefs, sexual orientation, the economic situation, and political situation in the home country (5, 6). Despite these extraordinarily stressful circumstances, it was still a difficult decision to leave. Often, an opportunity to flee was unplanned, and thus, the decision and the subsequent flee had to be made without adequate preparation (7). The opportunity for fleeing was frequently solicited by human traffickers. They were required to pay large sums of money or may be kidnapped and were required to pay a ransom to be able to leave their home country (7). Refugees are forced to travel with only a few personal belongings that they must carry (8). The journey can be very dangerous due to high risks of exploitation, rape, physical illness, and death by drowning or other injuries (9).

An asylum seeker is defined as a person who sought safety or prosperity by fleeing to another country and when arrived, officially asked for asylum which is mainly done at a refugee camp (5). When a person gets a temporary or permanent permit in the Netherlands, this person was identified as a “status holder” or “a person with a migration background” (10). In the Netherlands, refugee camps also known as asylum seeker centers are diverse. These centers provided only the very basic support, such as, “bed, bath, and bread.” Some asylum seekers required a more restricted environment due to the previous crime history. Unaccompanied minors were signed to small living facilities (10).

Asylum seekers in the Netherlands were considered a high-risk group for mental health problems. Adverse life events, forced migration, prolonged asylum procedures, and the complexity of the acculturation process can all contribute to higher levels of psychopathology (11). A study by Gerritsen et al. (12) revealed large variations in the prevalence of mental health problems. The prevalence of post-traumatic stress disorder (PTSD), depression, and anxiety vary from a few percent to more than 70% (12). In 2006, 28.1% of the asylum seekers from Afghanistan, Iran, and Somalia in Dutch asylum seekers centers suffered from PTSD and 68.1% suffered from depression or anxiety (13). Psychological issues are sometimes presented as physical symptoms which have a potential to mislead the health-care professional (14). Two research questions evolved: What are the threats to wellbeing in terms of mental health, psychosocial, and addiction problems, and what are the effective interventions for wellbeing for asylum seekers in asylum seeker centers in the Netherlands, reported in the literature?

## Methods

Due to the descriptive nature of this research, a scoping review was executed to identify influencing factors on the mental health of asylum seekers and to identify knowledge gaps in the literature (15). With a scoping review in a transparent and rigorous way, areas of research can be mapped (16). The Preferred Reporting Items For Systematic Reviews and Meta-analyses Extension for Scoping Reviews statement (PRISMA ScR) included recommendations in the design, literature search, analysis, and reporting of the scoping review (17). This descriptive study was registered in the open science framework registries (Registration DOI https://doi.org/10.17605/OSF.IO/YBR7F).

### Information Sources and Search

The search was conducted in the period before December 15, 2019 in the following databases: PsycINFO, Psychology & Behavioral Sciences Collection, Academic Search Premier, CINAHL MEDLINE, PubMed, Embase, and the Cochrane database. Additional articles were found using the snowballing process by searching through the reference list of previously identified articles.

Using the Problem Intervention Control Outcome (PICO) method, a search rule was constructed and an initial search was performed (18). From this search, initially, a few vital relevant articles were identified. The search rule was validated and improved by comparing the search terms with terms used in titles in the literature lists of vital articles. This resulted in new search terms which were added to the search rule. With this validated rule, the selected databases were searched again. All studies that met the inclusion criteria were uploaded into Rayyan R (19), a web application for systematic reviews.

### Search Items and Eligibility Criteria

The data were sought on the following variables “asylum seekers” OR refugees OR Exiles AND Netherlands OR Dutch, “effective measures” OR “real measures” OR “actual measures” OR “effective actions” OR “effective procedures” OR “effective methods” OR “effective processes” OR therapy OR Care OR prevention OR treatment OR “social support” AND “mental health” OR addiction OR “psychosocial problems,” wellbeing OR comfort OR welfare OR health OR safety (Appendix 1).

Included articles that met inclusion criteria reported threats on wellbeing in terms of mental health, psychosocial, addiction problems. Articles also included the effective interventions for wellbeing for asylum seekers in Dutch asylum seeker centers. English and Dutch publications were included. The following exclusion criteria were used: duplicate, newborn children, study protocol, abstract author, wrong population, diagnostic instrument, index, Switzerland, experience in Sweden, study design.

All studies that met the inclusion criteria were uploaded into Rayyan R and were ranked individually by FP and RE, blinded for each other. Afterward, studies that were not immediately agreed upon were discussed by FP and RE for a final decision.

### Critical Appraisal

To measure the quality of the identified articles, a critical appraisal for qualitative research from Treloar et al. (20) was used. For seven of the 10 key issues, the extent each selected study met the criteria set by Treloar et al. (20) (Appendix 2).

### Synthesis of Results

For identifying threats on wellbeing and interventions in the selected articles, we used the Neuman system model as its supports approaching the individual as an open system (21). This model is based on the combination of the stress coping, systems, and prevention theory, which helps in defining stressors and interventions. In this theoretical framework, wellbeing is defined as a continuum with wellbeing and illness at opposite ends of the continuum. Wellbeing for a person is associated with optimal system stability, which is the best possible state of wellbeing at any time (21). Threats for wellbeing are defined as barriers in dealing with stressors by insufficient possession of coping strategies and insufficient access to resources. Interventions are defined as support for dealing with stressors, support for increasing coping strategies, and access to resources. The data in the identified articles were categorized in the three means for self-efficacy stressors, coping strategies, and resources.

**Table:** 

**Figure 1:**
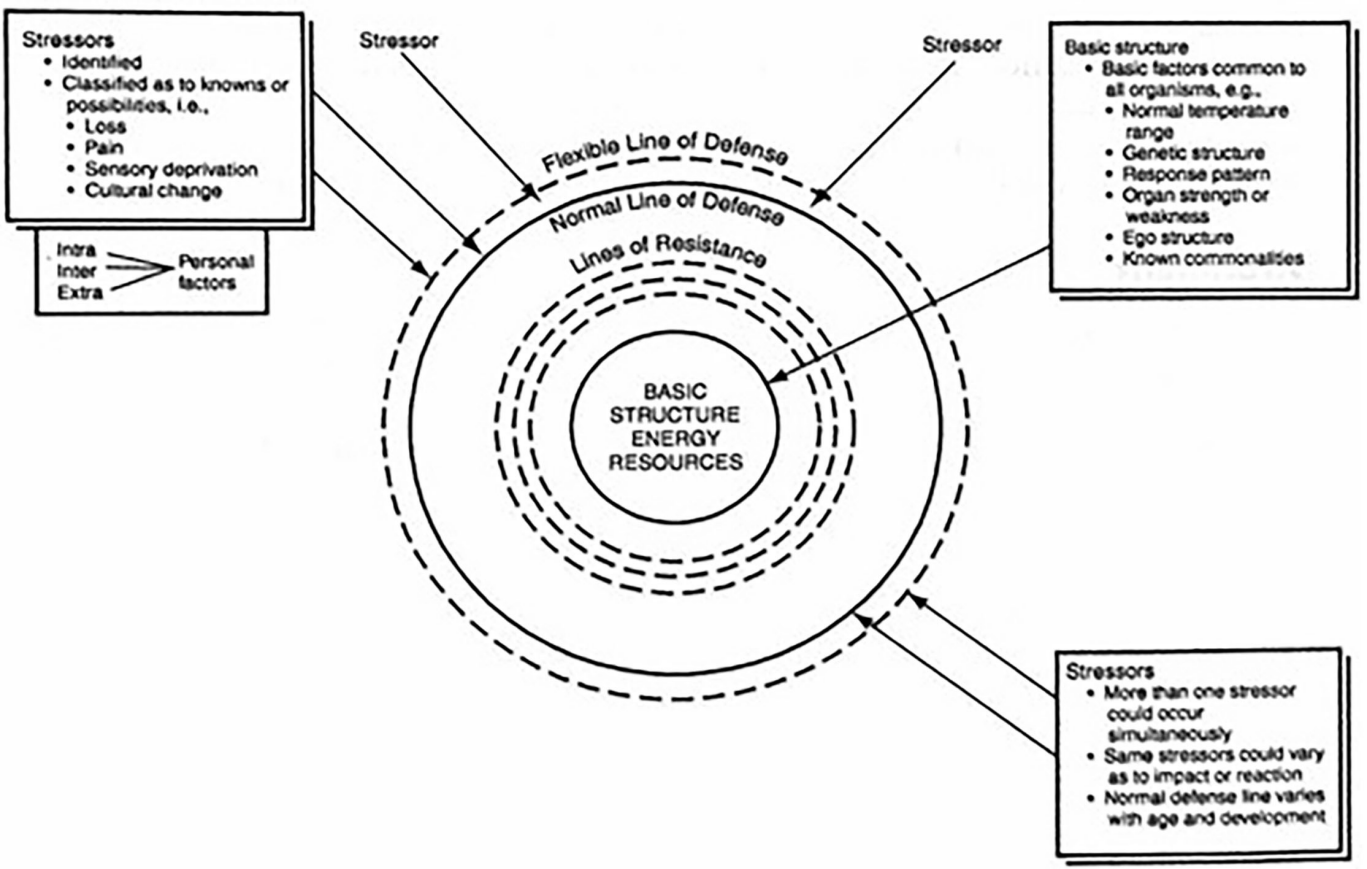
Essential parts of the NSM used for the syntheses of results. Reprinted from Neuman and Fawcett (21). Physiological, psychological, sociocultural, developmental, and spiritual variables occur and are considered simultaneously in each client concentric circle.

**Neuman system model**
In this framework, the client is seen as an open system illustrated by a series of concentric circles which Neuman has named the flexible line of defense, normal line of defense, and lines of resistance (refer to Figure 1). Each of these lines protect the client's basic structure, or central core from stressors in the environment. The circles and the core are composed of five related variables: physiological, psychological, sociocultural, developmental, and spiritual. The primary focus of the Neuman system model is the possibility for negative outcomes. This occurs when the person is not able to cope effectively with stressors from the environment (22). Each related variable provides a perspective on a person, how he or she grew up, the social economic situation and mental and physical condition. Furthermore, it provides insight on the view of the person in the present and the future. As such using these insights, severe illness and lower quality of life can be prevented.

#### Stressors

A tendency exists within any system to maintain a steady state or balance among the various disruptive forces operating within or upon it (23). The Neuman system model identifies these disruptive forces as stressors. Neuman system model environmental stressors are classified as intrapersonal between persons, interpersonal within the person, and extrapersonal outside the person in nature. They are present within as well as outside the client system. Intrapersonal stressors are internal environmental forces that occur within the boundary of the client system. Interpersonal stressors are external environmental interaction forces that occur outside the boundaries of the client system at a proximal range. Extrapersonal stressors are external environmental interaction forces that occur outside the boundaries of the client system at the distal range (21). In this study, the stressors were extracted to identify the threats to the wellbeing of the asylum seeker (Appendix 3).

#### Coping Strategies

According to Folkman and Lazarus, there is a specific role for care providers to mobilize client systems coping strategies: those refer to the specific efforts, behavioral and psychological, that asylum seekers use to master, tolerate, reduce, or minimize stressful events (24). Two general coping strategies are distinguished: problem-solving strategies and emotional-focused coping strategies. Problem-solving strategies are efforts to do something active to alleviate stressful circumstances, whereas emotion-focused coping strategies involve efforts to regulate the emotional consequences of stressful or potentially stressful events. Some coping strategies are active, and others are considered avoidant. Active coping strategies are either behavioral or psychological responses designed to change the nature of the stressor itself or how one thinks about the stressor. Avoidant coping strategies lead people into activities, such as alcohol use or mental states, such as withdrawal that keep them from directly addressing stressful events (21). In this study, skills were extracted that asylum seekers naturally have and that they have developed in their life to deal with stressful situations (Appendix 3).

#### Resources

The five dimensions of the client system function harmoniously in interactions with intrapersonal, interpersonal, and extrapersonal stressors. The extent of interactions between and among the five variables determines how much resistance a client system has to environmental stressors. Resources can be deployed on different variables. Physiological refers to the bodily structure and internal function. Psychological refers to mental processes and interactive environmental effects, both internally and externally. Sociocultural refers to the combined effects of sociocultural conditions and activities. Developmental refers to age-related development processes and activities. Spiritual refers to spiritual beliefs and influences. The resources are divided into the five variables that indicate in which the asylum seeker experiences support. This also provides insight into where resources are lacking among asylum seekers staying at asylum seeker centers, based on the data retrieved from the articles included in this scoping review (Appendix 3).

What influence intrapersonal, interpersonal, and extrapersonal stressors have on the wellbeing of the asylum seeker was extracted from the content of the included articles and subsequently described. Which coping strategies they already have or have learned from themselves or from others. We also describe which resources asylum seekers have or have access to during their stay at asylum seeker centers and these are subdivided into the five variables to provide insight into which threats there are and which interventions work well for this target group. Following the Neuman system model (Figure 1 and Appendix 3) concerning stressors, coping strategies, resources, and subthemes of these, we summarize the extracted data.

## Results

**Figure 2:**
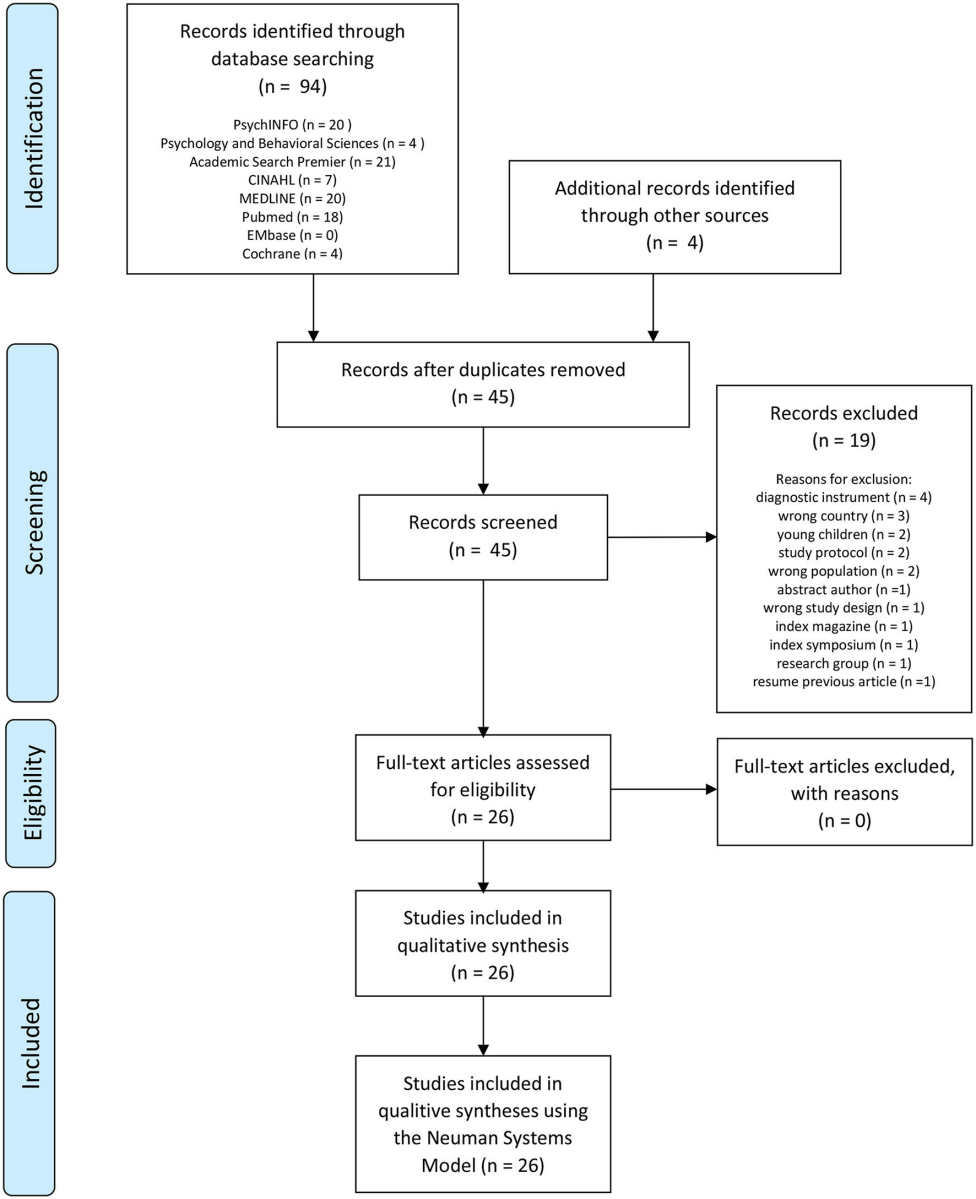
PRISMA flow diagram for the scoping review process.

In screening on domain and inclusion criteria in the databases and the additional snowball search, 94 records remained. After removing the duplicates, the database searches resulted in 45 citations. A total of 19 articles were excluded after reading the full text for the following exclusion reasons: diagnostic instrument, wrong country, young children, study protocol, wrong population, conference abstract only, wrong study design, index magazine, index symposium, and research group, which resulted in 26 articles included in this scoping review (Figure 2).

Following the Neuman system model, the retrieved data were reported on the topics: intrapersonal stressors, interpersonal stressors, extrapersonal stressors, coping strategies, and five types of resources.

### Stressors

More than one stressor may be imposed on the client system at any time (21).

#### Intrapersonal Stressors

The fact that asylum seekers were in an asylum procedure and therefore almost always are in a long-term stay in an asylum seeker center leads to the asylum seekers experience a lot of stress. This was also caused by uncertainty about the future and anxiety about being sent back to their country of origin or where they have previously reported in Europe (13, 25–31). Severely traumatized asylum seekers with long-lasting high levels of psychological burden often gave inconsistent interviews during the hearings of immigration services (25). Some asylum seekers said that the post-migration stressors, such as prolonged periods of uncertainty, fear, and boredom, cause more stress than pre-migration stressors (32). The access to medical and psychological care was generally difficult for asylum seekers with physical and psychological problems (25). As a consequence, there was a higher risk of suicide, especially in male asylum seekers, due to a lower use of mental health services and negative outcomes of their asylum procedure (27). Also, relationship issues, loss of family members, and other stressful life events were related to a higher rate of suicide in comparison with the Dutch population (27). In asylum seekers' children, the burden of frequent relocations was associated with an increase in mental health problems (33). Many asylum seekers who are mostly men report incidents of torture or other forms of torture (28). The prevalence rates of mental health and substance abuse disorders under undocumented asylum seekers were high (34). Unaccompanied adolescent asylum seekers who were staying in a special reception for first asylum registration in the Netherlands report more emotional problems and symptoms of anxiety (35). Also, the high number of incidents in the asylum seekers centers caused more emotional problems and symptoms of anxiety by minors (35). Asylum seekers experienced increasing post-traumatic stress due to their response to multiple post-traumatic experiences and ongoing post-migration difficulties. In summary, the major intrapersonal stressor “substance and drug abuse” of some asylum seekers triggers mental problems, gave physical complaints, and led to disruptive behavior among these asylum seekers inside and outside the asylum seekers center.

#### Interpersonal Stressors

The degree of burden by the experienced stress depends on various factors. The trauma experienced in the country of origin, during the trip, and living in an asylum seekers center in the Netherlands causes a major imbalance in daily life (13, 25, 26, 34, 36). Low health literacy of asylum seekers and their caretakers and cultural differences led to impaired communication between health professionals and asylum seekers (37). Loneliness, boredom, and shortage of money were major problems in asylum seekers living in asylum seekers centers. Some of them had started a distributive trade of beer, cannabis, khat, and opium to provide themselves with money and drugs to cope with these problems (26). Drug use is an underestimated problem in asylum seekers, and general practitioners should refer them to addiction care, which often does not occur (13, 30, 34). When asylum seekers were undocumented, they experienced often multiple adverse events or traumas, and the current life being undocumented was extra challenging (34). In summary, the major interpersonal stressors have been the influence of staying in the environment of an asylum seekers center with its tight regime, and scanty living conditions and involuntary living together with other asylum seekers from multiple countries with subsequent differences in cultural backgrounds, and speaking other languages, were very burdening for an individual living in such a center. Furthermore, the frequent relocations of asylum seekers interrupted the continuity of care and exposed the person to diverse suppressing environments in the different centers. They also lost social support which they had inside or outside the former center.

#### Extrapersonal Stressors

Frequent relocations between asylum seekers centers caused a lot of stress to asylum seekers due to the loss of all the familiar things at and around the asylum seekers center. Also, they cannot take all their personal belongings, so they had to buy these things again (31, 33, 37). It also caused a disruption of continuity of health care. In many cases, the loss or absence of medical records led to potentially life-threatening complications (37). Asylum seekers with physical complaints got more often asylum than asylum seekers with psychological symptoms (25). The migration of women from low-income to high-income countries increased the amount of maternal mortality and severe morbidity compared to host countries (38). Also, the increased level of popularity of anti-immigrant political parties in the Netherlands contributed to the vulnerability of women who were pregnant. The consequences of those political parties that were against immigrants had already resulted in lower reimbursement of interpretation services and significant cuts in health care (13, 32, 38). Asylum seekers suffered from uncertainty about their legal status and other resettlement stressors (31, 39). Asylum seekers did not have direct access to general practitioners but must be assessed and subsequently referred by a vocational trained nurse of the medical health center (13). Adequate and appropriate care should always be the main goal for healthcare professionals who worked with asylum seekers. However, there could be an ethical dilemma within those professionals in thinking that asylum seekers need to be treated differently (29). Because of the lack of money, undocumented asylum seekers had difficulties in accessing health care, and the expectation of limited treatment success discouraged the provision of mental health services (34). In summary, the major extrapersonal stressor had been health-care professionals and employees who did not detect or underestimate the underlying suffering of asylum seekers led to undertreatment and subsequent extra burdening.

### Coping Strategies

There was less to be found in the literature about what skills asylum seekers naturally have to cope with stress. Only two of the included articles provide intrinsic resources on how to deal with stress. The first article is specific about the use of substance abuse, some asylum seekers used substances to cope with loneliness others for boredom, but the majority used it to self-medicate for better sleep, less stress, and to deal with uncertainty and cope with mental health symptoms (26). The second article included learned skills that helped asylum seekers to cope with stress during this period. This concerns having self-confidence, a positive self-image, and having meaningful daytime activities, which creates perspective and ensures a feasible balance in their lives (29). Many organizations have tried to develop and gave preventive training for asylum seekers. This helped asylum seekers to develop active coping skills (40). Asking the asylum seekers what they needed to keep their mental health in balance in the uncertain state during the procedure could help to keep or reestablish a positive self-image (29). Healthcare providers can challenge patterns of coping by helping asylum seekers to see when their chosen coping styles are inadequate and when other strategies might work better (29).

Gaining refugee status in the Netherlands gave them restored feelings of safety and control over their life (31). Besides training of asylum seekers, there were several initiatives to improve the expertise of both medical and teaching staff on the asylum seekers centers (26).

### Resources

Resources were available for every asylum seeker if they know how to find these or had people who guide them to these resources. The first resources asylum seekers go to were usually located on the premises of the asylum seeker centers or near the asylum seeker centers and the people from the Central Agency for the Reception of Asylum Seekers, health center asylum seekers, or teachers in case of minors under the age of eighteen. The major assignment these professionals had is the prevention and treatment if necessary (13, 27, 38, 40). Available resources were instruments for assessing substance and drug abuse and health status. The RAR method is for assessing substance and drug abuse and the amount of drug usage in a population (26). The HIATUS tool provided for a standardized assessment in different dimensions, such as self-reported health and mental health status (41). Other resources included five interventions for treatment, therapy for PTSD, art therapy, education, cultural interview, and mindspring.

1. Therapy for asylum seekers diagnosed with PTSD in different mental health-care settings (31, 34, 36, 42)
2. Art therapy to help people to speak through art about their traumas if expressing themselves when words are limited (43)
3. Education focused on prevention as an intervention to learn about stressors, coping, and explain how to make use of help and healthcare sources (27, 44)
4. Cultural interview which supports gaining trust and recognizes the cultural background of the asylum seekers (45)
5. Mindspring: a community-based program in which trained asylum seekers give other asylum seekers in the asylum seeker centers group sessions on psychoeducation, psychosocial support, and empowerment (44).

#### Physiological

The physiological variable refers to the bodily structure and internal functioning (46). The general practitioner should proactively discuss the need for contraception (38). Many asylum seekers express their complaints as physical problems which in half of the cases cannot be physically diagnosed, and these complaints are sometimes associated with heightened stress (28). The general practitioner needed to be consulted and be informed by mental health professionals about how psychological problems could be missed when people present physiological problems (30). Healthcare workers needed to be aware of the effects on mental health by a strict reception policy on a reception center and provide this info to policymakers (35).

#### Psychological

The psychological variable refers to mental processes and interactive environmental effects both internally and externally (46). For more severe mental health problems, people were often referred to specialized care which was the institutions who are often not specialized in intercultural care and had no experience in care providing to people who speak other languages (13, 30, 32, 36, 47). Professionals should have considered culture as a key resource area in psychosocial wellbeing (32). Professionals used culturally informed interventions to understand the causes of psychological problems and interpretation by the asylum seeker crises are less severe (32). Better assessment to reveal common gaps in health information systems in Europe should formulate joint strategies and improve better access to health care for asylum seekers (41). Therapy in a group setting provided asylum seekers with holding, safety, and enlarged coping skills, and they could manage resettlement stressors in a more adequate way (31). Training professionals to recognize depression and suicidal behavior had shown massive effects on reducing suicide deaths in general populations and as well had resulted in asylums seekers population (27). In undocumented asylum seekers, it was helpful to offer a treatment program for those with psychiatric and addiction problems which gave them supportive and helpful contrast with the insecurities of daily life (34). Received treatment for PTSD after arriving in the Netherlands reduced the symptoms of PTSD. Consequently, it was important that general practitioners follow existing guidelines on quick referral to mental health care for asylum seekers who show PTSD symptoms (42). Psychoeducation and psychosocial support programs for asylum seekers who are trained by health-care professionals could have helped to raise awareness, give support, and could better understand the problems asylum seekers experience (44).

#### Sociocultural

The sociocultural variable refers to combined effects of sociocultural conditions and influences (46). Unaccompanied children received foster care from sometimes foster parents or the Nidos Foundation (40, 48). Groen (45) says that the cultural interview helps gaining trust and building an interrelationship by recognizing cultural roots. There was a need for cultural acceptable methods for family planning, especially under teenage girls as well as abortion (38). Some asylum seekers expressed their wish that a mental health professional should have helped them solve practical aspects, such as finding a job and arranging a residency permit (32). Language obstacles should have been recognized and solved using appropriate interpreter services (38). Access to medical health care was dependent on the interface of the characteristics of asylum seekers and their households, social and physical environments, and the characteristics of health systems, organizations, and providers (37). When asylum seekers were offered help in keeping and making meaningful contacts, they become engaged in activities (29). Besides the importance of the use of mental health care, it was important to improve contextual factors, such as employment, social, or family networks, becoming familiar with the new culture and social position during treatment (39, 42). To connect non-verbal with asylum seekers who suffered from severe problems, art therapy was a helpful way and showed what cannot be said (43).

#### Developmental

The developmental variable referred to age-related developmental processes and activities (46). School-based psychoeducation programs should have helped asylum seekers to develop active scoping skills to empower young asylum seekers and to help them cope with their trauma and stress problems. It was therefore extremely important that professionals gave asylum seekers training in psychological tools to have better active coping skills (40).

#### Spiritual

The spiritual variable refers to spiritual beliefs and influences (46). When some Islamic asylum seekers struggled to find help from mental health professionals, they often asked help from exorcist doctors because some are convinced that they suffer from the Jinn (49). Recognition of the legal status of asylum seekers gave them restored hope for the future (31). Some groups of asylum seekers, for example, Somali people, did not have much faith in Dutch health care (13). Asylum seekers should therefore be encouraged to find new identities by becoming caring parents, trustworthy friends, or members of a religious group which could have improved the quality of life (29). A major benefit from art therapy was that it creates space for unconscious processes (43).

## Discussion

Triggered by the reason for fleeing and the circumstances during their travel to the Netherlands, asylum seekers in Dutch asylum seekers centers experienced serious threats to their mental wellbeing. In 26 articles, the following threats were identified: the influence of staying in the environment of an asylum seekers center with its tight regime, scanty living conditions, and involuntary living together with other asylum seekers from multiple countries with subsequent differences in cultural backgrounds, and speaking other languages, substance and drug abuse among asylum seekers, health-care professionals and employees who do not detect or underestimate the underlying suffering of asylum seekers leading to undertreatment, and frequent relocations of asylum seekers. Two assessment instruments were identified: the RAR method and the HIATUS tool. Additionally, five interventions were identified: therapy for asylum seekers diagnosed with PTSD, art therapy, education focused on prevention as intervention, cultural interview, and mindspring.

The threats revealed that the care provided in the Dutch asylum seekers centers did not match the demand of asylum seekers sufficiently. This was also already reported in 2005 by Gerritsen et al. On the one hand, cultural aspects caused asylum seekers to not trust the health-care system in the Netherlands. On the other hand, the accessibility of health care was a problem for asylum seekers (13). For instance, asylum seekers did not have direct access to the general practitioner because health problems were usually assessed by a vocational trained nurse in the health center at the asylum seekers center. The provided care, which was frequently not matching the demand of the asylum seeker, started from the very beginning an asylum seeker enters the asylum seekers center (50).

Although this population was both characterized by a diverse cultural background and similarities in problems and care demand (51), there were, however, hardly any specific care programs available for these people (52). Despite all available mental health-care services, addiction care services, and the services from the health center at the asylum seekers centers, many health care workers did not have the specific required knowledge to work with this population. Although the RAR method is available, they were unaware of this method and they were also unaware of knowledge about the problem of drug abuse among asylum seekers (26). Due to the changes in refugee flow to the Netherlands, asylum seeker centers were closed and reopened (10). Subsequently, also, staff members change quickly which caused fewer opportunities for specialization and frequent loss of specialized knowledge had put pressure on adequate care and continuity of care. As a consequence, staff was not aware of available assessment instruments and interventions.

Some interventions were found in the literature, which are not applied in daily practice or in every asylum seekers center. Furthermore, there were also no actual protocols or guidelines in mental health care on the treatment of substance and drug abuse and addiction in this specific population (53). Yet in the literature, there were specific helpful assessment instruments and treatment interventions presented, as listed above. These were, however, often specific for a region or organization in the Netherlands and not widely integrated with care (52).

The frequent relocation from one to another asylum seekers center caused frequent interruption of continuity of care. The employees of the Central Agency for the Reception of Asylum Seekers do not always know an asylum seeker receives the care and if they do, they often do not inform the health-care professionals, both the former and new asylum seekers center (Personal communication, Operational manager Dutch health center for asylum seekers, 15-09-2020).

To appreciate these results, the issue of the limitation to the Dutch situation needs to be discussed. This scoping review was specifically aimed at the Dutch situation, so we may not have found stressors or interventions that were used in comparable situations in other countries in the literature. The political situation in the Netherlands and in the rest of Europe caused a negative influence on asylum policy. For example, there were cutbacks in (care) costs and there is a lot of resistance to the reception of asylum seekers in society. This caused a lower quality of life and more stressors for all asylum seekers.

## Conclusion

The knowledge on identifying and reducing threats, assessment, and treatment interventions for asylum seekers living in an asylum seekers center found in the literature provided perspectives on improving their wellbeing. The great diversity of cultural aspects and continuous changes in the number and origin of refugees in the Dutch asylum seekers centers disrupted the continuity of care. The absence of guidelines and required knowledge of professionals working with these asylum seekers led the question of what professionals need for providing effective health care to asylum seekers. Also, there is a need for insight into the amount of drug use and the accompanying number of drug problems in asylum seekers and the subsequent negative influence on their quality of life, health status, influence on disease, and interventions. The Netherlands was divided when it comes to the reception of asylum seekers. This was clearly visible in politics, media, and society. The result was a cost reduction in care for asylum seekers, which resulted in fragmentation in care, lower quality of care, and a higher burden of disease. The shortage of well-trained and intercultural expert healthcare personnel also caused loss of knowledge and the cessation of local or non-local interventions.

## Author Contributions

FP, RE, and EV contributed to the conception and design of the study. FP and RE reviewed the identified literature double-blind. FP wrote the first draft of the manuscript. All authors contributed to manuscript revision, read, and approved the submitted version.

## Funding

We greatly acknowledge Amethist Addiction Care (a non-profit organization) for making the hours available and the Master Integrated Care Design University of Applied Sciences Utrecht also for funding the open access publication.

## Conflict of Interest

The authors declare that the research was conducted in the absence of any commercial or financial relationships that could be construed as a potential conflict of interest.

## Publisher's Note

All claims expressed in this article are solely those of the authors and do not necessarily represent those of their affiliated organizations, or those of the publisher, the editors and the reviewers. Any product that may be evaluated in this article, or claim that may be made by its manufacturer, is not guaranteed or endorsed by the publisher.

## References

[1] IND and IND-BIC. Consulted From: Kerncijfers Asiel en Migratie. Ministry of Justice and Security Immigration and Naturalisation Service; IND Business Information Centre (2021). Rijksoverheid.nl

[2] Afghanistan: Taliban back in power. www.vluchtelingenwerk.nl. Consulted on 1 december 2021, van (2021). Available online at: https://www.vluchtelingenwerk.nl/feiten-cijfers/landen-van-herkomst/afghanistan (accessed December 01, 2021).

[3] WHO European Region. Health of Refugees and Migrants. (2018). Available online at: https://www.who.int/migrants/publications/EURO-report.pdf (accessed September 10, 2019).

[4] Braam R Dupont H Verbraeck H. Asielzoekers en middelengebruik; Een verkennend onderzoek naar middelengebruik en verslavingsproblematiek onder asielzoekers en een aanzet tot doelmatige voorlichting en interventie. (1999). Available online at: http://www.drugresearch.nl/media/88843-Asielzoekers%20en%20middelengebruik%20pdf.pdf (accessed September 10, 2019).

[5] Tribe R. Mental health of refugees and asylum seekers. Adv Psychiatr Treat. (2002) 8:240–8. 10.1192/apt.8.4.240

[6] Alessi EJ Kahn S Greenfield B Woolner L Manning D. A qualitative exploration of the integration experiences of LGBTIQQ refugees who fled from the middle east, north Africa, and central and south Asia to Austria and the Netherlands. Sexual Res Soc Pol J NSRC. (2018) 20:2018. 10.1007/s13178-018-0364-7

[7] UN refugee agency. Desperate Journeys - Refugees and migrants arriving in Europe and at Europe's borders - January – December 2018 - UNHCR. (2019). Available online at: https://www.unhcr.org/desperatejourneys/ (accessed July 1, 2019).

[8] Goldhill O. Photos: What Syrian Refugees Carry in Their Bags as They Leave Their Lives Behind. (2015). Available online at: https://qz.com/496220/photos-what-syrian-refugees-carry-in-their-bags-as-they-leave-their-lives-behind/ (accessed September 12, 2019).

[9] Braun-Lewensohn O Al-Sayed K. Syrian adolescent refugees: how do they cope during their stay in refugee camps? Front Psychol. (2018) 9:1258. 10.3389/fpsyg.2018.01258 30079046 PMC6063162

[10] COA. Sociale Veiligheid Van Bewoners in Asielzoekerscentra. Central Agency for the reception of Asylum Seekers. (2018). Available online at: https://www.coa.nl/sites/www.coa.nl/files/nieuws/media/bestanden/sociale_veiligheid_van_bewoners_in_asielzoekerscentra.pdf (accessed September 10, 2019).

[11] Laban C Dijk van R. Main topics in transcultural psychiatric research in the Netherlands during the past decade. Transcult Psychiatr. (2013) 50:792–816. 10.1177/1363461513503379 24071745

[12] Gerritsen AAM Bramsen I Devillé W Van Willigen LH Hovens JE Van der Ploeg HM. Health and health care utilisation among asylum seekers and refugees in the Netherlands: design of a study. BMC Public Health. (2004) 4:7. 10.1186/1471-2458-4-7 15070416 PMC385239

[13] Gerritsen AAM Bramsen I Devillé W Van Willigen LHM Hovens JE Van der Ploeg HM. Physical and mental health of Afghan, Iranian and Somali asylum seekers and refugees living in the Netherlands. Soc Psychiatr Psychiatr Epidemiol. (2005) 41:18–26. 10.1007/s00127-005-0003-5 16341619

[14] Heeren M Mueller J Ehlert U Schnyder U Copiery N Maier T. Mental health of asylum seekers: a cross-sectional study of psychiatric disorders. BMC Psychiatry. (2012) 12:114. 10.1186/1471-244X-12-114 22900706 PMC3490796

[15] Munn Z Peters MDJ Stern C Tufanaru C McArthur A Aromataris E. Systematic review or scoping review? Guidance for authors when choosing between a systematic or scoping review approach. BMC Med Res Methodol. (2018) 18:143. 10.1186/s12874-018-0611-x 30453902 PMC6245623

[16] Arksey H O'Malley L. Scoping studies: towards a methodological framework. Int J Soc Res Methodol. (2005) 8:19–32. 10.1080/1364557032000119616

[17] Tricco AC Erin L Wasifa Z O'Brien KK Colquhoun H Levac D. (2018). PRISMA extension for scoping reviews (PRISMA-ScR): checklist and explanation. Ann Internal Med. 2018:467–73. 10.7326/M18-0850 30178033

[18] Squires JE Valentine JC Grimshaw JM. Systematic reviews of complex interventions: framing the review question. J Clin Epidemiol. (2013) 66:13. 10.1016/j.jclinepi.2013.05.013 23953086

[19] Ouzzani M Hammady H Fedorowicz Z Elmagarmid A. Rayyan — a web and mobile app for systematic reviews. Syst Rev. (2016) 5:210. 10.1186/s13643-016-0384-4 27919275 PMC5139140

[20] Treloar C Champness S Simpson PL Higginbotham N. Critical appraisal checklist for qualitative research studies. Indian J Pediatrcs. (2000) 67:347–51. 10.1007/BF02820685 10885207

[21] Neuman B Fawcett J. The Neuman systems model. In: Neuman BM Fawcett J, editors, The Neuman Systems Model. 5th ed. Upper Saddle River, NJ: Pearson. (2011). p. 3–33.

[22] Gigliotti E Manister NN. A beginner's guide to writing the nursing conceptual model-based theoretical rationale. Nurs Sci Quart. (2012) 25:301–6. 10.1177/0894318412457060 23087334

[23] Skalski CA DiGerolamo L Gigliotti E. Stressors in five client populations: Neuman systems model-based literature review. J Adv Nurs. (2006) 56:69–78. 10.1111/j.1365-2648.2006.03981.x 16972920

[24] Folkman S Lazarus RS. An analysis of coping in a middle-aged community sample. J Health Soc Behav. (1980) 21:219. 10.2307/2136617 7410799

[25] Aarts R Wanrooij LV Bloemen E Smid G. Expert medico-legal reports: the relationship between levels of consistency and judicial outcomes in asylum seekers in the Netherlands. Torture J. (2019) 29:36–46. 10.7146/torture.v29i1.111205 31264814

[26] Dupont HJ Kaplan CD Verbraeck HT Braam RV Van de Wijngaart GF. Killing time: drug and alcohol problems among asylum seekers in the Netherlands. Int J Drug Policy. (2005) 16:27–36. 10.1016/j.drugpo.2004.06.002

[27] Goosen S Kunst AE Stronks K Van Oostrum IE Uitenbroek DG Kerkhof AJ. Suicide death and hospital-treated suicidal behaviour in asylum seekers in the Netherlands: a national registry-based study. BMC Public Health. (2011) 11. 10.1186/1471-2458-11-484 21693002 PMC3151232

[28] Hondius AJK Van Willigen LHM Kleijn WC Van der Ploeg HM. Health problems among Latin-American and middle-eastern refugees in the Netherlands: relations with violence exposure and ongoing sociopsychological strain. J Trauma Stress. (2000) 13:619–34. 10.1023/A:1007858116390 11109235

[29] Kramer S Bala J. Managing uncertainty; coping styles of refugees in western countries. Intervention. (2004) 2:33–42.

[30] Laban CJ Gernaat HBPE Komproe IH De Jong JTVM. Prevalence and predictors of health service use among Iraqi asylum seekers in the Netherlands. Soc Psychiatry Psychiatr Epidemiol. (2007) 42:837–44. 10.1007/s00127-007-0240-x 17676250 PMC2039804

[31] Droždek B Kamperman AM Tol WA Knipscheer JW Kleber RJ. Is legal status impacting outcomes of group therapy for posttraumatic stress disorder with male asylum seekers and refugees from Iran and Afghanistan? BMC Psychiatry. (2013) 13:148. 10.1186/1471-244X-13-148 23705873 PMC3665450

[32] Slobodin O Ghane S De Jong J. Developing a culturally sensitive mental health intervention for asylum seekers in the Netherlands: a pilot study. Intervention. (2018) 16:86. 10.4103/INTV.INTV_2_18

[33] Goosen S Stronks K Kunst AE. Frequent relocations between asylum-seeker centres are associated with mental distress in asylum-seeking children: a longitudinal medical record study. Int J Epidemiol. (2013) 43:94–104. 10.1093/ije/dyt233 24334208

[34] Lahuis AM Scholte WF Aarts R Kleber RJ. Undocumented asylum seekers with posttraumatic stress disorder in the Netherlands. Eur J Psychotraumatol. (2019) 10:1605281. 10.1080/20008198.2019.1605281 31231474 PMC6567193

[35] Reijneveld SA De Boer JB Bean T Korfker DG. Unaccompanied adolescents seeking asylum. J Nerv Ment Dis. (2005) 193:759–61. 10.1097/01.nmd.0000185870.55678.82 16260934

[36] Jongedijk RA Eising DD Van der Aa N Kleber RJ Boelen PA. Severity profiles of posttraumatic stress, depression, anxiety, and somatization symptoms in treatment seeking traumatized refugees. J Affect Disord. (2020) 266:71–81. 10.1016/j.jad.2020.01.077 32056948

[37] Baauw A Rosiek S Slattery B Chinapaw M Van Hensbroek MB Van Goudoever JB. Pediatrician-experienced barriers in the medical care for refugee children in the Netherlands. Eur J Pediatr. (2018) 177:995–1002. 10.1007/s00431-018-3141-y 29675644 PMC5997109

[38] Van den Akker T Van Roosmalen J. Maternal mortality and severe morbidity in a migration perspective. Best Practice Res Clin Obstetr Gynaecol. (2016) 32:26–38. 10.1016/j.bpobgyn.2015.08.016 26427550

[39] Sleijpen M Haagen J Mooren T Kleber RJ. Growing from experience: an exploratory study of posttraumatic growth in adolescent refugees. Eur J Psychotraumatol. (2016) 7:28698. 10.3402/ejpt.v7.28698 26886487 PMC4756627

[40] Bean T Eurelings-Bontekoe E Mooijaart A Spinhoven P. Factors associated with mental health service need and utilization among unaccompanied refugee adolescents. Admin Pol Mental Health Mental Health Serv Res. (2006) 33:342–55. 10.1007/s10488-006-0046-2 16755395

[41] Bozorgmehr K Goosen S Mohsenpour A Kuehne A Razum O Kunst A. How do countries' health information systems perform in assessing asylum seekers' health situation? Developing a health information assessment tool on asylum seekers (HIATUS) and piloting it in two European countries. Int J Environ Res Public Health. (2017) 14:894. 10.3390/ijerph14080894 28786927 PMC5580598

[42] Lamkaddem M Stronks K Devillé WD Olff M Gerritsen AA Essink-Bot ML. Course of post-traumatic stress disorder and health care utilisation among resettled refugees in the Netherlands. BMC Psychiatry. (2014) 14:90. 10.1186/1471-244X-14-90 24670251 PMC3986925

[43] Meijer-Degen F. Art therapy for mental health workers in areas affected by violence. Intervention. (2014) 12:99–107. 10.1097/WTF.0000000000000003

[44] Uitterhaegen B. Psycho-education and psychosocial support in the Netherlands; a program by and for refugees. Intervention. (2005) 3:141–7.

[45] Groen S. Recognizing cultural identity in mental health care: rethinking the cultural formulation of a somali patient. Transcult Psychiatr. (2009) 46:451–62. 10.1177/1363461509343087 19837781

[46] Neuman B Fawcett J. Neuman Systems Model. 4th ed. Hoboken, NJ: Prentice Hall (2002).

[47] Kramer SA Olsman E Hoogsteder MHH Van Willigen LHM. Sleepless nights because of ethical dilemmas in mental health care for asylum seekers. J Refug Stud. (2017) 31:466–87. 10.1093/jrs/fex039

[48] Zijlstra AE Menninga MC Van Os ECC Rip JA Knorth EJ Kalverboer ME. “There is no mother to take care of you.” Views of unaccompanied children on healthcare, their mental health and rearing. Environ Resident Treatm Child Youth. (2018) 36:118–36. 10.1080/0886571X.2018.1559118

[49] Raschke E. Immigrants and the Jinn. New York, NY: America Press Inc. (2017).

[50] Suurmond J Rupp I Seeleman C Goosen S Stronks K. The first contacts between healthcare providers and newly-arrived asylum seekers: a qualitative study about which issues need to be addressed. Public Health. (2013) 127:668–73. 10.1016/j.puhe.2013.04.004 23830729

[51] Rohlof H Knipscheer JW Kleber RJ. Use of the cultural formulation with refugees. Transcult Psychiatry. (2009) 46:487–505. 10.1177/1363461509344306 19837783

[52] Strijk PJM Van Meijel B Gamel CJ. Health and social needs of traumatized refugees and asylum seekers: an exploratory study. Perspect Psychiatr Care. (2010) 47:48–55. 10.1111/j.1744-6163.2010.00270.x 21418072

[53] Jong JTVM Dijk RCJ. Handboek culturele psychiatrie en psychotherapie (Tweede geheel herziene druk). Utrecht: De Tijdstroom (2020).

