# Supplementary material for: Threats and Interventions on Wellbeing in Asylum Seekers in the Netherlands: A Scoping Review
Source: Front Psychiatry. 2022 Apr 1;13:829522. doi: 10.3389/fpsyt.2022.829522 (PMC9010677; doi:10.3389/fpsyt.2022.829522)
Supplement: Supplementary file 1 [file Data_Sheet_1.pdf]

Appendix 1

Search rule ((AB "asylum seekers" OR AB refugees OR AB Exiles) AND (AB Netherlands OR AB Dutch) AND ((AB "effective measures" OR AB "real measures" OR AB "actual measures" OR AB "effective actions" OR AB "effective procedures" OR AB "effective methods" OR AB "effective processes" OR AB therapy OR AB Care OR AB prevention OR AB treatment OR AB "social support") AND (AB "mental health" OR AB addiction OR AB "psychosocial problems")) AND (AB wellbeing OR AB comfort OR AB welfare OR AB health OR AB safety))

Table search overview in the different databases										
Threats on well-being in terms of mental health, psychosocial and addiction problems and effective interventions for well-being for asylum seekers in asylum seeker centers in the Netherlands			PsychINFO	Psychology and Behavioral Sciences Collection	Academic Search Premier	CINAHL	MEDLINE	Pubmed	EMbase	Cochrane
	Keywords	Search strings	Date 14-12-2019	Date 14-12-2019	Date 14-12-2019	Date 15-12-2019	Date 15-12-2019	Date 13-12-2019	Date 15-12-2019	Date 15-12-2019
P:	Asylum seekers in the Netherlands	("asylum seekers" OR refugees OR Exiles) AND (Netherlands OR Dutch)	140	34	461	53	161	166	1.476	6 trials
I:	Threats and effective interventions	(("effective measures" OR "real measures" OR "actual measures" OR "effective actions" OR "effective procedures" OR "effective methods" OR "effective processes" OR therapy OR Care OR prevention OR treatment OR "social support") AND ("mental health" OR addiction OR "psychosocial problems"))	103.424	19.133	75.921	46.029	83.341	104.738	503.860	351 reviews<br>10 protocols<br>21348 trials<br>9 editorials<br>2 special collections<br>2 clinical answers
O:	Well-being	(wellbeing OR comfort OR welfare OR health OR safety)	597.708	152.158	1.776.391	748.465	1.865.813	2.275.875	7.718.093	479 reviews<br>541 protocols<br>399531 trials<br>103 editorials<br>21 special collections<br>29 clinical answers
P+I			21	4	22	8	22	75	0	6 trials
P+I+O			20	4	21	7	20	18	0	4 trials

Appendix 2

Table: Methodological Appraisal of the include articles							
Key issues in critical appraisal of qualitative research	Clear purpose of the study	Appropriate rationale	clear outline of the conceptual framework	Clarity methods of data analysis and study purpose addressed	Clarity treats to reliability and validity in data collection, analysis and interpretation	Clear progression from research question to conclusions	Overall judgment methodological quality
Aarts et al 2019 * <br>Expert medico-legal reports. The relationship between levels of consistency and judicial outcomes in asylum seekers	+	+	+	+	+	+	good
Akker & van Roosmalen 2015 <br>Maternal mortality and severe morbidity in a migration perspective	+	±	±	-	-	±	mediocre
Baauw et al 2018 <br>Pediatrician-experienced barriers in the medical care for refugee children in the Netherlands	+	+	+	+	+	+	good
Bean et al 2006 <br>Factors Associated with Mental Health Service need and Utilization among Unaccompanied Refugee Adolescents	+	+	+	+	+	+	good
Bozorgmehr et al 2017 <br>How Do Countries' Health Information Systems Perform in Assessing Asylum Seekers' Health Situation? Developing a Health Information Assessment Tool on Asylum Seekers (HIATUS) and Piloting It in Two European Countries	+	±	±	+	±	+	mediocre
Drozdek et al 2013	+	+	+	+	+	+	Good

Is legal status impacting outcomes of group therapy for posttraumatic stress disorder with male asylum seekers and refugees from Iran and Afghanistan?							
Dupont et al 2005<br><br>Killing time drug and alcohol problems among asylum seekers in the Netherlands	+	+	+	+	+	+	Good
Gerritsen et al 2006<br><br>Use of health care services by Afghan Iranian and Somali refugees and asylum seekers living in The Netherlands	+	±	±	+	+	+	Good
Gerritsen et al 2006<br><br>Physical and mental health of Afghan, Iranian and Somali asylum seekers and refugees living in the Netherlands	+	+	+	+	+	+	Good
Goosen et al 2011<br><br>Suicide death and hospital-treated suicidal behaviour in asylum seekers in the Netherlands a national registry-based study	+	+	+	+	+	+	Good
Goosen et al 2014<br><br>Frequent relocations between asylum-seeker centers are associated with mental distress in asylum-seeking children: a longitudinal medical record study	+	+	+	+	+	+	Good
Groen 2009<br><br>Recognizing cultural identity in mental health care rethinking the cultural formulation of a Somali patient	+	+	-	±	±	+	Mediocre
Hondius et al 2000<br><br>Health Problems Among Latin-American and Middle-Eastern Refugees in the Netherlands Relations With Violence Exposure Sociopsychological Strain	+	+	+	+	+	+	Good

Jongedijk et al 2020<br><br>Severity profiles of posttraumatic stress, depression, anxiety, and somatization symptoms in treatment seeking traumatized refugees	+	+	+	+	+	+	Good
Kramer & Bala 2004<br><br>Managing uncertainty; coping styles of refugees in western countries	+	+	±	+	±	+	Good
Kramer et al 2017<br><br>Sleepless Nights because of Ethical dilemmas in mental health care for asylum seekers	+	±	±	+	+	+	Good
Laban et al 2007<br><br>Prevalence and predictors of health service use among Iraqi asylum seekers in the Netherlands	+	+	+	+	+	+	Good
Lahuis et al 2019<br><br>Undocumented asylum seekers with pots traumatic stress disorder in the Netherlands	+	+	+	±	+	+	Good
Lamkaddem et al 2014<br><br>Course of post-traumatic stress disorder and health care utilization among resettled refugees in the Netherlands	+	+	+	+	+	+	Good
Meijer 2014<br><br>Art therapy for mental health workers in areas affected by violence A rarely explored resource	±	±	+	±	-	+	Mediocre
Raschke 2017<br><br>Immigrants and the Jinn	-	+	-	-	-	-	Low

Reijneveld et al 2005<br><br>Unaccompanied Adolescents Seeking Asylum Poorer Mental Health Under a Restrictive Reception	+	+	±	+	±	+	Good
Sleijpen et al 2016<br><br>Growing from experience an exploratory study of posttraumatic growth in adolescent refugees	+	+	+	+	+	+	Good
Slobodin et al 2018<br><br>Developing a culturally sensitive mental health intervention for asylum seekers in the Netherlands a pilot study	+	+	+	+	+	+	Good
Uitterhaegen 2005<br><br>Psycho-education and psychosocial support in the Netherlands; a program by and for refugees	±	±	+	±	-	+	Mediocre
Zijlstra et al 2018<br><br>There is no mother to take care of you Views of unaccompanied children on healthcare their mental health and rearing environment	+	+	+	+	±	+	Good

Appendix 3

Table: overview data of included studies					
First author, year, , title	Stressors	Coping strategies	Resources	Summary points	Narrative
Aarts et al, 2019<br>Expert medico-legal reports<br>The relationship between levels of consistency and judicial outcomes in asylum seekers	Trauma<br>Refugee status<br>Extensive research to prove trauma	-	Mental health care<br>Other organizations involved	Severely traumatized asylum seekers and thus psychologically disturbed give inconsistent information during their first asylum hearings and thereby lowering the change of obtaining asylum. Mental health problems cause longer procedure then by a physical condition. But also depends on the person who takes the hearings. Asylum procedures are often long and complex. Especially the lack of access to extensive examination in psychological signs and symptoms of trauma is causing underdiagnosis. They advise to place more medical professionals at asylum seeker centers.	Stressors as multi trauma in the past cause extra stressors during hearings since psychologically disturbed give inconsistent information during their first asylum hearings and thereby lowering the change of obtaining asylum. Extra specialist professionals and access to extensive examination in psychological signs and symptoms of trauma are external resources to decrease the inconsistencies.
Akker & van Roosmalen 2015<br>Maternal mortality and severe morbidity in a migration perspective	Higher risk at maternal mortality due to complications during pregnancy and childbirth	-	Prevention by health care professionals	Maternal mortality and severe morbidity in asylum seekers occur more often as compared to host countries. They have an extended risk due to a different risk profile. The problem is in general the asylum seekers have no knowledge about the health system in the host country. Also cultural, linguistic, or social-economic barriers may lead to life - threatening delays in providing sufficient health care. Beside this risk, due to increasing popularity of anti-immigrant parties, may lead to increasing vulnerability of migrant pregnant women. An intervention such as education on the asylum seekers center may lead to better understanding of access to health care in the host country.	Lack of knowledge about the health system in the host country and cultural, linguistic, or social-economic differenced leads to insufficient coping systems leading to barriers to healthcare. Education on the asylum seekers center as an external resource may lead to better understanding of access to health care in the host country.
Baauw et al 2018<br>Pediatrician-experienced barriers in the medical care for refugee children in the Netherlands	Increased burden of mental and physical health<br>Frequent relocations<br>Unknown medical history<br>Poor handoffs of medical records<br>Poor health literacy<br>Cultural differences	-	-	Refugee children are at risk for an increased burden of physical and mental health conditions. They experience many barriers to access health care. The most important one is frequent relocations this leads to the lack of continuity of care. This is the result of policies and regulations and causes potentially life-threatening complications due to unknown medical history and loss of medical records. Access to health care is at the interface between the characteristics of person households, social en physical environments and the characteristics of health systems, organizations and providers. They also points out that the main obstructions, such as frequent relocations, unknown	Stressors as frequent relocations, leading to lack of continuity of care and barriers to care which cause extra stressors such as increased burden of physical and mental health conditions not seldom causing life threatening complications. No coping and no resources are reported.

Table: overview data of included studies					
First author, year, , title	Stressors	Coping strategies	Resources	Summary points	Narrative
				and incomplete medical history and poor handoffs, leads to impaired communication between health care professionals.	
Bean et al 2006<br>Factors Associated with Mental Health Service need and Utilization among Unaccompanied Refugee Adolescents	Psychological stress<br>Lack of policies on adolescents	-	Nidos Foundation<br>Teachers<br>Health care providers	Unaccompanied refugee minors concerning their mental health and wellbeing. Environment and living conditions, especially on asylum seekers centers, are major stressors to unaccompanied refugee minors. Caregivers have an important role to support them. Therefore, the have be trained. Also, school can help to provide them as save place to cope with the consequences of trauma and stress. Their advice is that school and mental health care work together to provide help to these unaccompanied refugee minors.	Trauma and stress due to living on an asylum seekers center cause major stress to unaccompanied refugee minors. The support should be provided in collaboration with school and mental health care providers.
Bozorgmehr et al 2017<br>How Do Countries' Health Information Systems Perform in Assessing Asylum Seekers' Health Situation? Developing a Health Information Assessment Tool on Asylum Seekers (HIATUS) and Piloting It in Two European Countries	-	-	The use of the health information assessment tool (HIATUS) when entering host country	A health information assessment tool on asylum seekers in two different countries in the EU to check the health information systems: This tool provides a standardized assessment in different dimensions such as self-reported health and mental health status.	In The health information assessment tool on asylum seekers helps to reveal gaps in access to health care for asylum seekers in the Netherlands and Germany.
Drozdek et al 2013<br>Is legal status impacting outcomes of group therapy for posttraumatic stress disorder with male asylum seekers and refugees from Iran and Afghanistan?	Legal status (asylum seeker/refugee), living arrangements (living alone/living with family/living as an incomplete family), housing (living in an asylum seeker center/common housing), family left behind (spouse/children/parents/other relatives), work (income through paid job/welfare), fluently Dutch language.<br>Major losses and life changes related to family life, legal status and living arrangements.	-	Group therapy about PTSD symptoms in mental health institutes	The influence that legal status has on asylum seekers who have group therapy for post-traumatic stress disorder. causes stressors also due being an asylum seeker in The Netherlands and due to the resettlements between the different centers. Getting a legal change, the perfective on the future and giving the asylum seeker more control on their lives. More longitudinal research have to be done to find out which factors gives asylum seekers the most stress.	Relocations, living conditions and lack of support are threats on well-being which cause a lack of control which influence the outcome of group trauma therapy. On the other hand, getting a legal status gives perspective and a feeling of being in control.

Table: overview data of included studies					
First author, year, , title	Stressors	Coping strategies	Resources	Summary points	Narrative
Dupont et al 2005<br>Killing time drug and alcohol problems among asylum seekers in the Netherlands	Nature and development of drug use among refugees. Drug use may complicate their application to permanent refugee status.<br>Crisis in home country, their flight and the insecure situation in the Netherlands were associated with drug and alcohol use.<br>Cultural background and expectations associated with drugs.	Drug and alcohol use to kill time, kill memories and uncertainty during the long procedure.	RAR method	Asylum seekers experience a lot of empty days waiting on their decision of the procedure to get a legal status. During that time, they have no opportunity to work or study after the age of 18. This is a stressor which cause that people search for other coping strategies such as drugs abuse. It also helps to cope with trauma related symptoms.	Asylum seekers use drugs to cope with boredom, waiting on a decision on their legal status and cope with traumatic experience. The RAR method could help to assess these problems and the amount of drugs usage in this population.
Gerritsen et al 2006<br>Use of health care services by Afghan Iranian and Somali refugees and asylum seekers living in The Netherlands	-	-	Health services use	There are different aspects in which asylum seekers make use of health care services. Cultural background, trustworthy and assessments done by the general nurse who is assessing the condition of the asylum seeker and decides if the asylum seeker can have an appointment with the general practitioner.	The health care system did not change much in the past years. The accessibility of health care is a problem for asylum seekers. Also, cultural aspects make that asylum seekers do not trust the health care system in The Netherlands.
Gerritsen et al 2006<br>Physical and mental health of Afghan, Iranian and Somali asylum seekers and refugees living in the Netherlands	Uncertain future and obtaining a permit. Recent history of traumatic experiences living in reception centers and not allowed to work.	-	Prevention and treatment	Physical and mental health problems are highly prevalent among asylum seekers due to uncertainty about their future, traumatic experiences and don't have distraction during the day such as work.	Asylum seekers experience a lot of physical and mental health problems caused by traumatic experiences. Beside that the living conditions and not being allowed to work is not helping in coping with these problems. Also, uncertainty about their future is a major stressor.
Goosen et al 2011<br>Suicide death and hospital-treated suicidal behavior in asylum seekers in the Netherlands a national registry-based study	Burden of suicide and hospital-treated suicidal behavior. Asylum procedure. Relation issues. Loss of a family member. Transfer between centers. Substance abuse. Living conditions.	-	Prevention	Traumatic experiences, asylum procedure, loss of family, transfer between centers, substance abuse and living conditions could lead to suicidal thoughts and behavior. This is in most cases related to mental health issues. Males are at higher risk, but they use mental health services less. Training physicians in recognizing and treating depression and suicidal behavior shown impressive effects in reducing suicide death rates.	Traumatic experiences, asylum procedure, loss of family, transfer between centers, substance abuse and living conditions could lead to suicidal thoughts and behavior. This is in most cases related to mental health issues. Males are at higher risk, but they use mental health services less. Training physicians in recognizing and treating depression and suicidal behavior shown impressive effects in reducing suicide death rates.
Goosen et al 2014<br>Frequent relocations between asylum-seeker centres are associated with mental distress in asylum-	Mental distress by relocations in children. Differences in reception centers as well as in health care provision.	-	Minimizing extra support for relocated children especially in vulnerable	Frequent relocations is related to mental distress in asylum seeking children especially in children who are vulnerable. The relocations cause a disruption in the continuum of care. Health care providers and	Children and especially vulnerable children experience mental distress due to frequent relocations. Health care providers should be aware and monitoring asylum seeking children if extra care is needed.

Table: overview data of included studies

First author, year, , title	Stressors	Coping strategies	Resources	Summary points	Narrative
seeking children: a longitudinal medical record study			children. Relocations contribute to the prevention of mental distress.	others who are involved should be aware and monitoring whether these children need extra care.	
Groen 2009 Recognizing cultural identity in mental health care rethinking the cultural formulation of a Somali patient	-	-	Cultural interview. Gaining trust and recognizing cultural roots. Interrelationship	The cultural interview could gain trust and recognizing cultural background of the asylum seekers from in this case a Somali patient.	The cultural interview greatly improves the relationship with an asylum seeker and is important to create a basis for treatment.
Hondius et al 2000 Health Problems Among Latin-American and Middle Eastern Refugees in the Netherlands Relations With Violence Exposure Socio Psychological Strain	Not trusting the interpreters. Busy with legal procedures or relocations.	-	Primary health care.	Asylum seekers report many complaints at the medical health center on the reception center. Mostly due to mental distress but also because of the situation they are in. The waiting and uncertainty of the procedure and frequent relocations causes mental and psychical problems. Due to not trusting the interpreter’s people’s needs are often not met.	Attention should be paid to past violent experiences in relation to complaints which asylum seekers address to the general practitioner. The extent to which translators are trusted by the asylum seekers is something to take into account when diagnosing the patient's complaints.
Jongedijk et al 2020 Severity profiles of posttraumatic stress, depression, anxiety, and somatization symptoms in treatment seeking traumatized refugees	PTSD	-	Treatment at ARQ centrum ’45.	Many refugees have severe post migration symptoms of posttraumatic stress, depression, anxiety and somatization. In this third echelon of care organization existing treatment programs are helpful for trauma related disorders and comorbid disorders.	Specialized care in the Netherlands for asylum seekers have treatment programs for asylum seekers with trauma related and comorbid disorders.
Kramer & Bala 2004 Managing uncertainty; coping styles of refugees in western countries	Uncertainty	Self-image, social contacts, activities, perspective and balance	-	From the perspective of the experience of asylum seekers, mental health could be improved. Improving and re-establishing a positive self-image, meaningful contacts en strengthening and help find new identity.	Health care providers can challenge patterns of coping by helping asylum seekers find out if there coping styles are adequate. Also re-establishing positive self-image and have meaningful contacts helps strengthening the asylum seekers.
Kramer et al 2017 Sleepless Nights because of Ethical dilemmas in mental health care for asylum seekers	Lack of information and communication problems to access mental health.	-	Professional standards regarding diagnosis and treatment for professionals.	Health care professionals need a guideline providing a framework on inherent ethical dilemmas. Also, shared values in treatment program and organizations helps professionals. Efforts should be made by al involved to be respectful and provide adequate and appropriate care.	Guidelines and frameworks help health care professionals to cope with ethical dilemmas in their work with asylum seekers so that they be respectful and provide adequate and appropriate health care.
Laban et al 2007 Prevalence and predictors of health service use among	Long asylum procedure. Psychopathology. Cooperation between primary and	-	Health services	This paper shows that there is a huge number of unmet need for mental health care in Iraqi asylum seekers. This is due to the Dutch medical health	There is a lower use of health services by Iraqi asylum seekers due to the differences in health care systems between country’s due to the fact

Table: overview data of included studies					
First author, year, , title	Stressors	Coping strategies	Resources	Summary points	Narrative
Iraqi asylum seekers in the Netherlands	secondary care in mental health, often it stagnates in primary.			system which is different to the Iraqi health care system. Also, cooperation between primary and secondary care is not optimal and leads to undertreatment and lower trust and therefore lower use of health services.	they can't go directly to a medical specialist. Also, this causes a lack of trust in health care professionals.
Lahuis et al 2019 Undocumented asylum seekers with pots traumatic stress disorder in the Netherlands	Challenging social circumstances. Adverse events and trauma'. Barriers in access to health care due to being undocumented.	-	Treatment for undocumented asylum seekers with PTSD	One year program for undocumented asylum seekers with PTSD to stabilize, give trauma focused therapy (TFT) and let them orientate on their future. Beside this professionals should be trained with a specific attitude towards undocumented asylum seekers.	When asylum seekers are undocumented and coping with PTSD they face a lot of challenging social circumstances. Because they are undocumented they don't have access to health care services due to not being insured for health care costs. There are a few organizations which help the undocumented with their extremely stressors and need of health care. So UAS developed and researched a health care program which consists stabilization, evidence based TFT and a future oriented module.
Lamkaddem et al 2014 Course of post-traumatic stress disorder and health care utilisation among resettled refugees in the Netherlands	PTSD, the flight, delay in application for a permit, loneliness	-	Mental Health care and PTSD treatment	If asylum seekers with mental health follow a treatment for PTSD, they have less symptoms later on. Asylum seekers who don't have treatment symptoms increase or have late onset symptoms of PTSD. Beside that in addition to mental health care it is important to integrate focus on employment, social/family networks, becoming familiar with the new culture, and social position.	Mental health treatment is effective on PTSD, but it depends on primary health care providers to follow guidelines and refer asylum seekers to mental health providers as soon as possible. Beside mental health treatment focusses to improve contextual factors is very important to improve mental health of asylum seekers.
Meijer 2014 Art therapy for mental health workers in areas affected by violence A rarely explored resource	``	-	Art therapy, nonverbal therapy	Art therapy training helps professionals to create a nonverbal way of communication with people from various backgrounds. It also creates room for unconscious processes. Patients' art can show us what is unable to talk about.	Art therapy is a way to communicate nonverbal and patients can show trough art what they ate not able to talk about.
Raschke 2017 Immigrants and the Jinn	Fleeing from war and brutality, political situation in the Netherlands (2014), struggle to find mental health	Practices of exorcism to target demons called Jinn	Exorcist doctors	Immigrants use practices exorcism to cope with mental health issues. Related to Islam demons called Jinn.	In the Netherlands Islamic refugees sometimes choose to use exorcism to cope with mental health problems to target demons which called Jinn.
Reijneveld et al 2005 Unaccompanied Adolescents Seeking Asylum Poorer Mental Health Under a Restrictive Reception	Stringent reception policy, riots, fights, emotional problems, anxiety,	-	-	Stringent reception policy causes emotional problems under unaccompanied adolescents asylum seekers. Mostly they present symptoms of anxiety and the longer they stay takes symptoms increase Health care professionals should be aware of the effects of the strong reception policy in this target group and inform policy makers about these effects.	Health care professionals should be aware of anxiety symptoms due to a strong reception policy on unaccompanied adolescent asylum seekers.

Table: overview data of included studies					
First author, year, , title	Stressors	Coping strategies	Resources	Summary points	Narrative
Sleijpen et al 2016<br>Growing from experience an exploratory study of posttraumatic growth in adolescent refugees	Traumatic events, PTSD symptoms, forced flight, war zone presence, exposure to combat action, loss of close family member or friend	Perceived social support, satisfaction with live, autonomy	-	Post traumatic growth due to potentially traumatic events can cause a fundamental reconfiguration of a person's life goals. Living in an asylum seekers center in the Netherlands can cause potentially traumatic events. Post traumatic growth was unrelated to PTSD symptoms but positively to satisfaction with life. Social support and dispositional optimism were positively related to post traumatic growth.	Asylum seekers can experience post migration traumatic events causing post traumatic growth and a reconfiguration of life goals. This is not related to PTSD symptoms. Social support could help redefining these goals.
Slobodin et al 2018<br>Developing a culturally sensitive mental health intervention for asylum seekers in the Netherlands a pilot study	Loss of identity, feeling of uncertainty, helplessness, ambivalence towards mental health problems		Local support by increasing knowledge caregivers	Mental health and psychosocial support has become a common future in emergency humanitarian responses. The pilot study was to investigate asylum seekers needs and expectations in terms of mental health to allow the development of a culturally sensitive intervention.	Mental health and psychosocial support adds on the local support of mental health. Culturally sensitive interventions should be integrated in mental health care. Mental health and psychosocial support is not strictly European but could be helpful at any humanitarian emergency.
Uitterhaegen 2005<br>Psychoeducation and psychosocial support in the Netherlands; a program by and for refugees	Causes of flee, travel to Netherlands, lack of social/family support, uncertainty of future, loss of positive social identity.		Asylum seekers trained in psycho education to help others. Mindspring.	A community-based program within the asylum seekers population on an asylum seekers center: Trained asylum seekers giving group sessions on psychoeducation, psychosocial support and empowerment.	Asylum seekers and refugees are trained to help others to raise awareness on trauma, stress, feelings of guilt, acculturation, alcohol and drugs abuse. Also, they teach to cope with those problems.
Zijlstra et al 2018<br>There is no mother to take care of you Views of unaccompanied children on healthcare their mental health and rearing environment	Emotional problems, environment, age when arriving in host country	Peer contacts, education, high quality rearing environment.	Guardian for unaccompanied children, foster parents	The importance of monitoring the health of unaccompanied children after arriving in the host country: Needs of this population changes due to changes in flight streams, policy and characteristics of the population. Health professionals need to try to create a bond with these children.	Professionals working with minor asylum seekers should first gain trust and bond with these children. This leads to better monitoring their health en mental well-being.
